# Developments in Pb-210 methodologies to provide chronologies for environmental change

**DOI:** 10.1007/s10653-022-01215-x

**Published:** 2022-03-22

**Authors:** H. N. Hunter, C. J. B. Gowing, A. L. Marriott, J. H. Lacey, S. Dowell, M. J. Watts

**Affiliations:** 1 Inorganic Geochemistry, Centre for Environmental Geochemistry, British Geological Survey, Nottingham, NG12 5GG UK; 2 Stable Isotope Facility, Centre for Environmental Geochemistry, British Geological Survey, Nottingham, NG12 5GG UK; 3 University of Surrey, Guildford, GU2 7XH UK; 4 University of Plymouth, Plymouth, PL4 8AA UK

**Keywords:** Pb-210, Cs-137, Anthropocene, Chronology, CRS, rPlum, Lake sediment

## Abstract

Chronologies generated from core profiles to apply dates to environmental changes commonly use the measurement of the activity of radionuclides deposited and stratified with physical environmental material. The most commonly reported nuclide to define chronologies covering the last 150 years is Pb-210, for which accepted data processing methodologies in the literature have focussed on the constant rate of supply (CRS) model and the more recently published Bayesian Plum model. This short communication describes a validation approach using defined sediment layers referred to as ‘varve’ counting, which provide known points of reference to account for uncertainty between generated dates from each model using published Pb-210 measurements. A significant improvement in the chronologies was observed when applying reference date corrections to the models. This was shown to be essential in providing confidence in reported datasets and accuracy of predicted chronologies, which will better inform the interpretation of environmental change, e.g. sedimentation rates, climate change, pollution pathways and land degradation. Generated chronologies from both the CRS and Plum methods showed good agreement with the established varve dates (typically < 4-year difference).

## Introduction

Largely spearheaded by Appleby and Oldfield (Appleby, 2001; Appleby & Oldfield, 1978; Appleby et al., 1988), Pb-210 dating presented an opportunity to provide a chronology for environmental changes over the past 150 years (Appleby, 2001), especially when used alongside other dating techniques, such as tephrochronology, carbon-14, and analytical techniques such as ICP-MS to track changes in sediment composition over time. The past 150 years incorporates a period of rapid acceleration in historical anthropogenic activities, such as construction, agriculture, mining and fossil fuel consumption. With increasing awareness of the influence of anthropogenic activities on human, animal and ecosystem health and the possibility for having to address legislative restrictions, the importance of dating the chronology of events is required to identify past trends and timelines to inform management strategies for the legacy of past anthropogenic activities and to inform the forecasting of future trends for environmental change and subsequent mitigation or adaptive strategies.

Other established methods for dating geological records are used to calculate ages in the range of hundreds to thousands of years using pollen records (Wang et al., 2019), ash layers (tephrochronology) and carbon-14 (Pontevedra-Pombal et al., 2018) and in the range of millions to billions of years for uranium-lead isotope ratios (Parrish, 2013). The extrapolation of data and information from such measurement techniques is unsuitable when attempting to model chronologies for more recently deposited environmental materials covering the acceleration of anthropogenic activities. The use of Pb-210 measurements to define chronologies of environmental deposition (e.g. sedimentation) is the most suitable method for the time period spanning the last 150 years (Appleby, 2001; Barsanti et al., 2020) and is particularly suited to measurement by gamma spectrometry (Appleby et al., 1988). Since gamma spectrometry is a non-destructive technique, often limited sample mass can be re-used for other analytical measurements.

A pivotal point in attempts to refine Pb-210 dating techniques was the publication of the constant initial concentration (CIC) method and the constant rate of supply (CRS) method (Appleby & Oldfield, 1978). The CRS method has since become the most widely used method for modelling Pb-210 measurements to define chronologies (Aquino-López et al., 2018; Barsanti et al., 2020). More recently, Aquino López et al. (2020) reported the Plum model, which was built upon the CRS model, creating a statistical model based on Pb-210 flux (Aquino-López et al., 2018). Notwithstanding these developments, there have been very few ways to objectively compare and contrast these methods, beyond the use of man-made datasets intended to replicate sediment cores (Aquino-López et al., 2018; Tylmann et al., 2016). Therefore, the validation and standardisation of methods associated with modelling Pb-210 measurements are essential, in particular when considering the variations of the CRS method reported in the literature (Barsanti et al., 2020; Smith, 2001).

In both the Plum and CRS models, corrections can use defined historical occurrences such as the position of the maximum Cs-137 activities within the sedimentary column. These are attributable to the peak of atomic weapons testing in 1963 (Appleby, 2001) and/or the Chernobyl accident in 1986 (Appleby, 2001; Bjerregaard, Andersen and Andersen, 2015), allowing calculated ages to be informed by having one or more sediment layers corrected to these reference dates. However, the presence of the Cs-137 activity maxima is not always detectable, particularly in areas unaffected by the fallout from the Chernobyl accident (Appleby, 2001). Techniques such as tephrochronology or significant changes in pollen records (Blais et al., 1995) may be viable additions or alternatives to Cs-137 maxima for the purposes of providing a reference date. However, these techniques require specific circumstances, which will not be applicable to every core so cannot be used consistently.

**Fig. 1:**
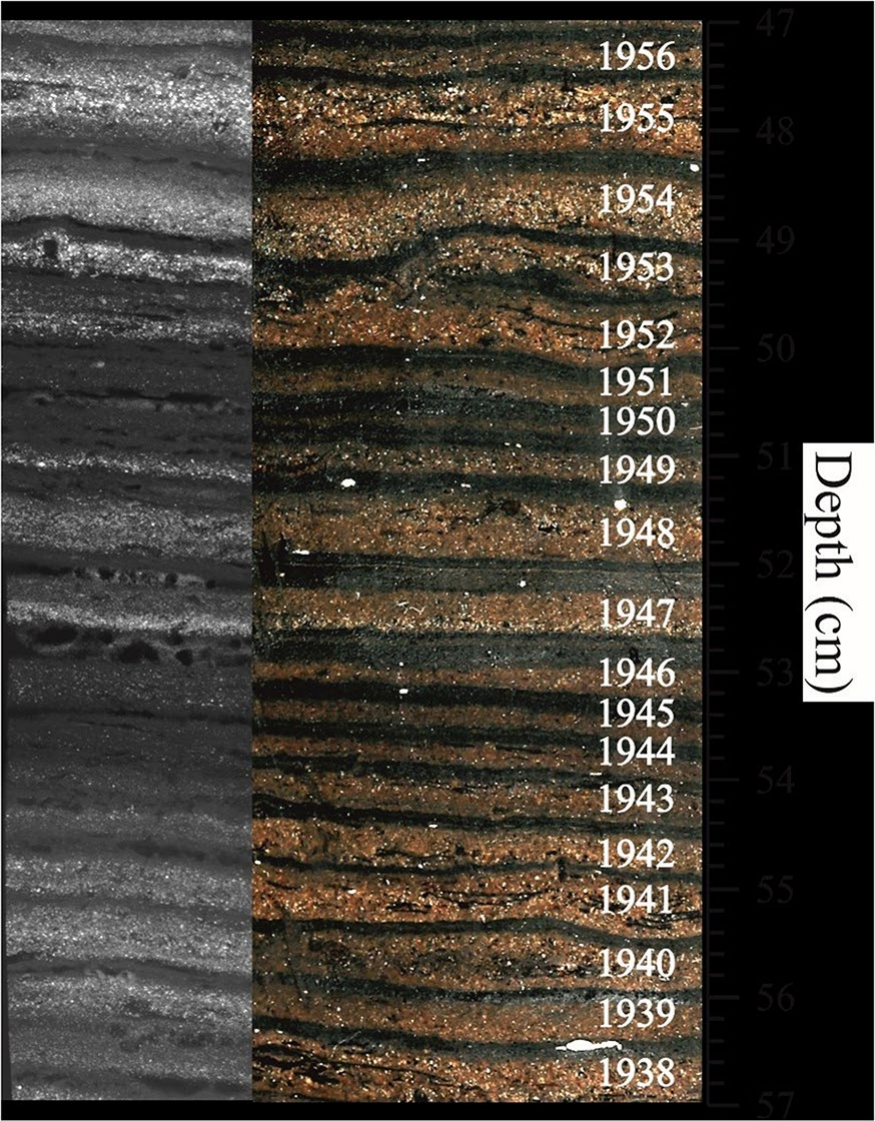
An image of the KEVO-1 and KEVO-3 freeze cores from Lake Kevojärvi. (Haltia et al., 2021, Fig. 4) © A.-P. Leppänen

Haltia et al. (2021) recently published the use of cores from Lake Kevojarvi with clearly defined layers (varves) attributed to annual snowmelt cycles (Haltia et al., 2021), with lighter layers denoting the sediment washed into the lake by the annual snowmelt and dark layers representing sediment formed, while the snow is frozen (Fig. 1). This provides a rare opportunity for method validation (Sanchez-Cabeza & Ruiz Fernández, 2012; Tylmann et al., 2016). By relating these cycles to calendar dates, as illustrated in Fig. 1, and then comparing to the acquired Pb-210 and Cs-137 data presented in Haltia et al. (2021), both the CRS and Plum methods of Pb-210 dating can be applied to provide an independent validation of the results produced by the two techniques.

## Pb-210 dating calculations

**Fig. 2:**
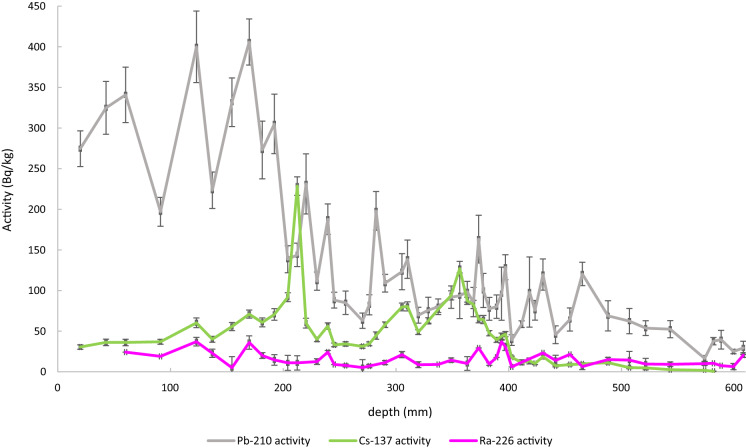
Activity of unsupported Pb-210, Cs-137 and Ra-226 in dry weight for each varve plotted against depth (data from E. Haltia et al., 2021). Error bars represent 1σ uncertainty

Haltia et al. (2021) reported core data from the KEVO-1 freeze core for which nuclide activity and physical data are summarised (Haltia et al., 2021; Tables 1 and 2). For the purpose of this short communication, data published by Haltia et al. (2021) have been reprocessed using four approaches: two making use of the CRS method and two making use of the Plum method. The CIC method was originally intended to be included in the analysis, but was omitted from the comparison due to the unsupported Pb-210 data not following a simple logarithmic pattern with depth (Fig. 2). For each method, age calculation was initially carried out using only Pb-210 (for total supported and unsupported Pb-210 activity) and Ra-226 (to determine the supported Pb-210 activity) data, and then, a second age calculation was completed using Pb-210, Ra-226 and Cs-137 activity data (Fig. 2) to refine the models. For the purpose of method comparison, the ages given by the varves within the sediment were assumed to be definitive.

**Fig. 3:**
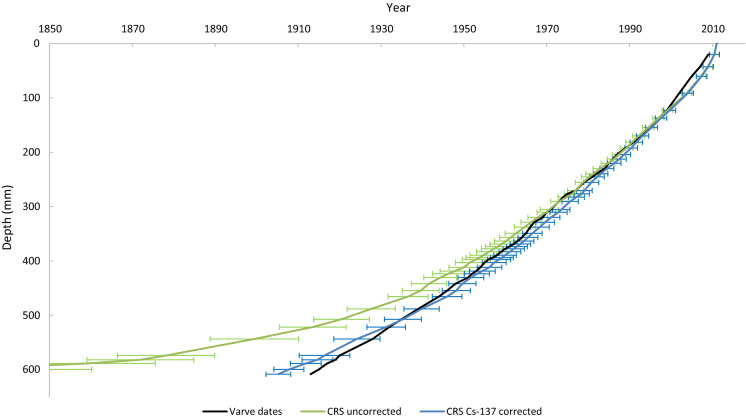
CRS age depth profile with and without Cs-137 correction and varve dates

The data provided in Tables 1 and 2 of Haltia et al. (2021) were first processed to calculate inventories and ages with associated uncertainties by applying the equations listed in Appleby (2001), specifically Eqs. 18, 28, 29, 36, 42 and 45, in Microsoft Excel™. This was then cross-referenced against the data provided in Table 3 of Haltia et al. (2021) to ensure the consistency of the method. The same data were subsequently processed using the freely available rBacon (v 2.5.5) and rPlum (v 0.2.1) packages for RStudio (RStudio 2021), following the guide provided by Aquino-López (2020) and Blaauw and Christen (2021), from which the raw data output was transferred from RStudio into Microsoft Excel for clear presentation. Figures 3 through 6 are created using the output from these two methods and the two datasets. In Plum, the maximum and minimum values calculated by the Bayesian process for each depth were used to create an uncertainty range.

**Fig. 4:**
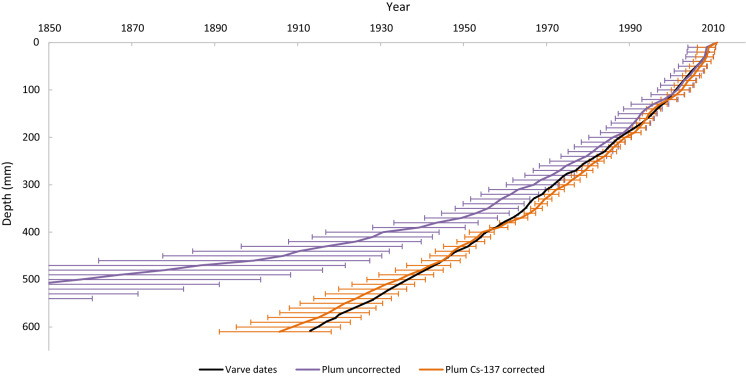
Plum age depth profile with and without Cs-137 correction and varve dates

Models using simply Pb-210 and Ra-226 activity showed appreciable deviation from the varve ages, which may be explained by the core being too short to represent the full unsupported Pb-210 profile (Haltia et al., 2021). Models that included a Cs-137 correction were more closely matched the actual age of sediment layers (Figs. 3 and 4). Appleby (2001) noted that the accuracy of Pb-210 ages could be verified by comparison to, or improved by, incorporation of the Cs-137 activity profile that may be applied to determine the age of a sediment layer (Blais et al., 1995), which may explain why ages derived using such methods were relatively more accurate, allowing those models to operate as if a full Pb-210 profile had been measured.

The uncorrected models follow the same pattern as their corrected counterparts, but with a bias towards older ages that increases with depth, particularly when looking at samples deeper than 400 mm for the Plum model and 500 mm for the CRS model. The deviation with depth is largely due to the way the age is calculated, as indicated using the CRS model, which assumes that the unsupported Pb-210 activity reaches zero immediately past the last given activity, which is in this case the lowest measured sample depth. Due to the Pb-210 profile being incomplete in this dataset, this is a false assumption within the calculation, and the Cs-137 corrected models can be used to predict the unsupported Pb-210 remaining beneath the point at which the core data ends (Appleby, 2001).

This deviation can be demonstrated by the CRS model equation (Eq. 1), which shows as A approaches zero, t increases at an increasing rate and hence the age becomes increasingly biased.

*Equation 1. Calculation of sediment age using the CRS model (*Appleby & Oldfield, 1978*, Eq. 9)*

$$t=\frac{1}{\uplambda }\times \mathrm{ln}\frac{A(0)}{A}$$

wheret is the age of the sediment layer in years;λ is the Pb-210 decay constant,A(0) is the total unsupported Pb-210 inventory of the core; and.

A is the unsupported Pb-210 inventory below the sediment layer.

The correction using the position of the maximum Cs-137 activity can be illustrated by considering the CRS equation being modified by inclusion of a coefficient estimated from the known age and Pb-210 inventory of the layer with the maximum Cs-137 activity (Eq. 2).

*Equation 2. Illustrative adaptation of the CRS calculation (after *Appleby & Oldfield, 1978*, Eq. 9)*

$$t=\frac{1}{\uplambda }\times \mathrm{ln}\frac{\left(A\left(0\right)+c\right)}{\left(A+c\right)}$$

As the values of A(0) and A are increased by an equal amount (c), the logarithmic term decreases and by extension so does the calculated age and the bias on that age. This has a more significant effect on sediment layers closer to the bottom of the core, where A is very small, leading to the differences illustrated in Figs. 3 and 4. This clearly demonstrates the need to have a core which extends far enough to capture the entire unsupported Pb-210 inventory, or a measured maximum Cs-137 activity (or other independent age indicator) to avoid the appreciable bias.

In this instance, the core was incomplete (610 mm) and the full Pb-210 profile was not included within the data. Consequently, the methods that did not include the Cs-137 correction deviated from the established profile and can be disregarded (Smith, 2001).

**Fig. 5:**
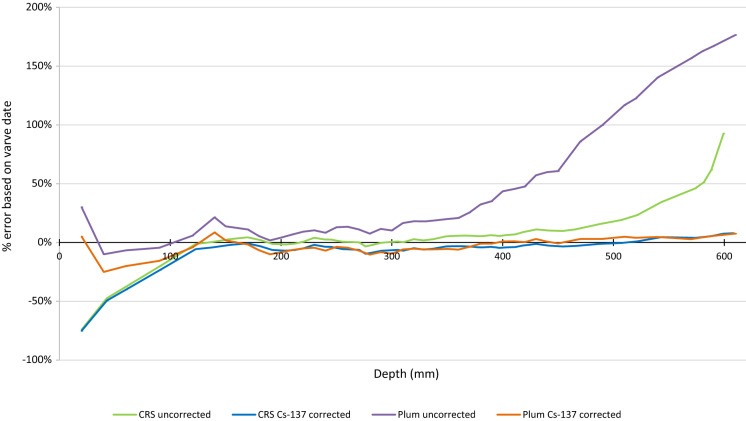
Per cent error of each age measurement compared to the closest varve depth

It should be noted that when provided with an accurate measure of the core’s sedimentation rate, the uncorrected Plum model considerably outperformed the uncorrected CRS model in the lower sections of the core. This would have produced a set of ages, which closely resembled the Cs-137-corrected chronology with an average negative bias of 9% below 400 mm, where it previously deviated far more significantly (bias of 44–176% from depths 410 to 610 mm) (Fig. 5). However, as this would require an existing chronology to calculate the sedimentation rate, which would not typically be available, the data have been omitted, and the default sedimentation rate within the Plum model’s settings was used instead. Furthermore, reliance on an existing chronology would introduce bias favouring results similar to the previous calculations, which may have concerning implications should there be an unseen error in the previous chronology used to calculate the sedimentation rate.

## Discussion

The calculated ages using both the CRS and Plum models align closely to each other and differ from the definitive varve ages by less than 4 years down to the 90-year boundary, beyond which the difference increases to a maximum of 8 years (CRS) and 7 years (Plum). Both models provide accurate estimates of the deposition dates when compared to the definitive varve dates.

**Fig. 6:**
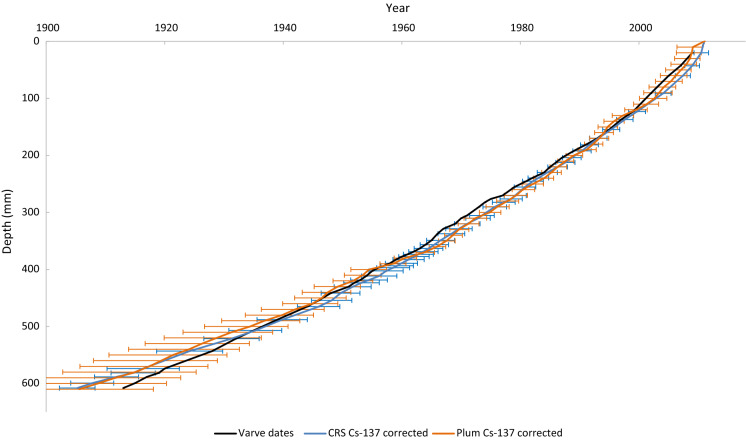
Comparison of the CRS and Plum model ages against varve ages

The Plum model tends to generate ages with a greater uncertainty than the CRS model (e.g. + 10, -12 years (Plum), ± 4 years (CRS) at 580 mm depth), particularly towards the end of the core (Fig. 6). Though this can be presented as a detrimental feature, it may be more realistic in certain situations (Aquino-López et al., 2020), despite this example having a low degree of sediment mixing, illustrated by clearly visible and well-defined varves (Fig. 1), demonstrating that the sedimentary layers are undisturbed, this is not always the case. Without a clear indicator, it is difficult to determine the degree to which two sediment layers may have mixed, especially in a core with a low sedimentation rate, for example, where a 10-mm section may contain several years-worth of compacted sediment. In cases such as the above, uncertainty may be regarded in a similar manner to a confidence interval showing the range of years of sedimentation, which make up a core section. It is also notable that at the bottom of the core the varve dates are within the Plum model’s uncertainty range but not that of the CRS model, uncertainty for which was calculated following equations of Appleby (2001).

Though based on the same principles as the CRS model (Aquino-López et al., 2018), the Plum model’s age/depth profile does not follow a logarithmic curve to the same degree, indicating that it is more reliant on the Pb-210 activity of the individual samples taken from the core. Notably at 570 mm one sample, measured with an unexpectedly low activity, was omitted due to significantly older ages being calculated in the core preceding the 570 mm depth, whereas the CRS model was unaffected by the sample inclusion or omission. It is notable that the two models compare more favourably to each other than the varve ages which, though close, appear to follow a more linear path. Considering the similarity of the two models, it is likely that this is largely due to the limitations of the data provided to the models as opposed to error within the models themselves.

The use of the positions of the layers with maximum Cs-137 activities (or other reference dates) is critical in ensuring valid results are calculated from an incomplete Pb-210 profile (Barsanti et al., 2020; Sanchez-Cabeza & Ruiz Fernández, 2012; Smith, 2001; Tylmann et al., 2016). However, care must be taken not to rely on this method. Haltia et al. (2021) observed that the maximum Cs-137 activity, which would be associated with the 1963 nuclear weapons testing, was within the varve assigned to 1964, suggesting that although 1963 was identified as the year when atmospheric levels of Cs-137 were at their highest, the material did not settle out (at least in this region) until 1964. This small difference in date may introduce error when using the maximum Cs-137 as an estimate to compensate for not having a complete Pb-210 inventory in other cores, even though in this case it could be accounted for.

Another factor to be considered is that nuclides can be mobile between sediment layers (Haltia et al., 2021). Even in the core used by Haltia et al. (2021), where sediment layers appeared pristine, Cs-137 was observed in sediments deposited prior to the first nuclear tests conducted in 1945 (Fig. 2, where depths below approx. 460 mm are pre-1945). This has no effect on the use of Cs-137 maximum activities to determine reference dates as the maximum activities will remain the same. However, as described by Haltia et al. (2021), in this core Cs-137 was observed to be more mobile in the sediment than other nuclides such as Pb-210 (Alonso González, 2015; Appleby et al., 1988). Therefore, Cs-137 should only be used to identify maximum activities associated with the 1963 nuclear weapons and 1986 Chernobyl accident.

## Conclusions

While both the CRS and Plum models were able to produce chronologies, which convincingly follow the varve ages of the sediment layers, without reference dates they deviate with unacceptable bias chronologies at depths further down the core. It has been demonstrated that, in cores under similar conditions to the KEVO-1 freeze core, chronologies will experience bias without the application of independent reference dates and if possible the use of a dataset containing samples from all depths up to the end of unsupported Pb-210 within the sediment. Barsanti et al. (2020) observed “it is essential to compare the Pb-210 chronology with some independent temporal markers to validate the age model”. Though correct, due to the inconsistency of such markers’ availability, it is important that future Pb-210 dating models are not reliant on such data to be able to produce accurate chronologies. Ideally reference ages would only be required to confirm chronologies produced by Pb-210 data. In future, considering the ease of use of each of the CRS and Plum methods, there would be an advantage in making use of both methods as agreement between multiple chronologies can increase the confidence in the interpretation of measured data.

As the Plum method and the technology behind it were relatively new compared to the CRS method, it is likely that capabilities will develop further, and models like Plum will be able to process information specific to each core. When paired with advancements in equipment such as improved detectors for gamma spectrometers, future chronologies will be more detailed than those currently available. As Pb-210 dating models and gamma spectrometers are developed further and improved, methods of comparison, such as the use of the cores described in Haltia et al. (2021), Sanchez-Cabeza and Ruiz Fernández (2012) and Tylmann et al. (2016) as reference datasets, should be encouraged and developed alongside new developments in Pb-210 dating procedures in order to demonstrate confidence and traceability in the measurements and the subsequent interpretation of the data.

Overall, the similarity between the CRS and Plum models is clear, and therefore, it is likely, with the data provided and the assumption of a constant rate of supply required by both models (Appleby, 2001; Aquino-López et al., 2018), that in this instance any deviation between the calculated Pb-210 chronologies and the definitive varve dates is due to the limitations of the data acquired rather than the models themselves. Though it has been demonstrated that using reference dates as a correction does not fully eliminate the difference between the Pb-210 chronologies and the definitive varve dates. Chronologies generated by the Plum and CRS models are acceptably accurate to be applied when informing the tracking of environmental change.

## Data Availability

Software code is open-access rPlum.

## References

[CR1] Alonso González, M. (2015) Cantabrian estuary sediment analysis by gamma espectroscopy of 210Pb and 137Cs: sedimentation rate, dating and biodiffusion effects. Available at: http://hdl.handle.net/10902/7116 [Accessed online 22nd July 2021].

[CR2] Appleby P (2001). Chronostratigraphic techniques in recent sediments. Tracking environmental change using lake sediments.

[CR3] Appleby PG, Oldfield F (1978). The calculation of lead-210 dates assuming a constant rate of supply of unsupported 210Pb to the sediment. CATENA.

[CR4] Appleby PG, Nolan PJ, Oldfield F, Richardson N, Higgitt SR (1988). 210Pb dating of lake sediments and ombrotrophic peats by gamma essay. Science of the Total Environment.

[CR5] Aquino-López MA, Blaauw M, Christen JA, Sanderson NK (2018). Bayesian analysis of 210Pb dating. Journal of Agricultural, Biological and Environmental Statistics.

[CR6] Aquino-López MA, Ruiz-Fernandez AC, Blaauw M, Joan-Albert Sanchez-Cabeza J-A (2020). Comparing classical and Bayesian 210Pb dating models in human-impacted aquatic environments. Quaternary Geochronology.

[CR7] Barsanti M, Garcia-Tenorio R, Schirone A, Rozmaric M, Ruiz-Fernandez AC, Sanchez-Cabeza JA, Delbono I, Conte F, De Oliveira Godoy JM, Heijnis H, Eriksson M, Hatje V, Laissaoui A, Nguyen HQ, Okuku E, Al-Rousan SA, Uddin S, Yii MW, Osvath I (2020). Challenges and limitations of the 210Pb sediment dating method: Results from an IAEA modelling interlaboratory comparison exercise. Quaternary Geochronology.

[CR8] Bjerregaard, P., Andersen, C. B. I. & Andersen, O. (2015) Ecotoxicology of metals—sources, transport, and effects on the ecosystem. In: Nordberg, G. F., Fowler, B. A., & Nordberg, M. (eds) *Handbook on the toxicology of metals (Fourth Edition)*. Fourth Edi. San Diego: Academic Press, pp. 425–459

[CR9] Blaauw, M., J. & Christen, J. A. (2021) *Plum: Main 210Pb age-depth modelling function*. Available at: https://rdrr.io/cran/rplum/man/Plum.html [Accessed: 3 June 2021].

[CR10] Blais JM, Kalff J, Cornett RJ, Evans RD (1995). Evaluation of 210Pb dating in lake sediments using stable Pb, Ambrosia pollen, and 137Cs. Journal of Paleolimnology.

[CR11] Haltia E, Leppänen A-P, Kallio A, Saarinena T (2021). Sediment profile dating and reconstructing nuclear events from annually laminated lake sediments in northern Finland. Journal of Environmental Radioactivity.

[CR12] López, M. A. A. (2020) *PAGES-ECR-rPlum*, *PAGES-ECR-rPlum*. Available at: https://github.com/maquinolopez/PAGES-ECR-rPlum- [Accessed: 3 June 2021].

[CR13] Parrish R, Rink WJ, Thompson J (2013). Uranium–Lead dating. Encyclopedia of scientific dating methods.

[CR14] Pontevedra-Pombal X, Castro D, Souto M, Fraga I, Blake WH, Blaauw M, López-Sáez JA, Pérez-Díaz S, Valcárcel M, García-Rodeja E (2018). 10,000 years of climate control over carbon accumulation in an Iberian bog (southwestern Europe). Geoscience Frontiers.

[CR15] Sanchez-Cabeza JA, Ruiz Fernández AC (2012). 210Pb sediment radiochronology: An integrated formulation and classification of dating models. Geochimica Et Cosmochimica Acta.

[CR16] Smith JN (2001). Why should we believe 210Pb sediment geochronologies?. Journal of Environmental Radioactivity.

[CR17] Tylmann W, Bonk A, Goslar T, Wulf S, Grosjean M (2016). Calibrating 210Pb dating results with varve chronology and independent chronostratigraphic markers: Problems and implications. Quaternary Geochronology.

[CR18] Wang Y, Goring SJ, McGuire JL (2019). Bayesian ages for pollen records since the last glaciation in North America. Scientific Data.

